# Pro370Leu mutant myocilin disturbs the endoplasm reticulum stress response and mitochondrial membrane potential in human trabecular meshwork cells

**Published:** 2007-04-19

**Authors:** Lina Wang, Yehong Zhuo, Bingqian Liu, Shengsong Huang, Fei Hou, Jian Ge

**Affiliations:** State Key Laboratory of Ophthalmology, Zhongshan Ophthalmic Center, Sun Yat-sen University, Guangzhou, China

## Abstract

**Purpose:**

To investigate the impact of Pro370Leu mutant myocilin on endoplasmic reticulum (ER) stress response and mitochondria function in human trabecular meshwork (HTM) cells.

**Methods:**

HTM cells were transfected with wild-type Pro370Leu mutant myocilin or pcDNA3.1 (+) expression plasmids. The effect of the mutant myocilin on ER stress response was semiquantitatively evaluated by determining the expression level of 78 kDa glucose-regulated protein (GRP78) using reverse transcription-polymerase chain reaction and phosphorylation of the α subunit of eukaryotic translation initiation factor 2 (eIF2α) using western blot analysis. Mitochondria function was determined by analyzing the changes in mitochondrial membrane potential (*Δψm*), measured by flow cytometry analysis using the fluorescent probe JC-1.

**Results:**

Pro370Leu mutant myocilin attenuated the induction of GRP78 and the phosphorylation of eIF2α. In HTM cells expressing the mutant myocilin, the reductions were evident in the level of GRP78 mRNA (65.5±2.0%), GRP78 protein (22.5±2.3%), and eIF2α phosphorylation (30.6±2.6%), compared to cells transfected with the wild-type myocilin plasmid (p less than or equal to 0.05). There was no significant difference between wild-type-myocilin- and pcDNA3.1(+)-transfected cells. Furthermore, Pro370Leu mutant myocilin caused a collapse of *Δψm* in HTM cells.

**Conclusions:**

Pro370Leu mutant myocilin down-regulates the ER stress response and destroys the *Δψm* of HTM cells. These observations suggest that Pro370Leu mutant myocilin could affect ER and mitochondria function through a gain of function, increasing its vulnerability to various cellular injuries and producing dysfunctional HTM cells.

## Introduction

Glaucoma, characterized by progressive optic neuropathy, is the second most common blinding disease in the world [1]. Primary open angle glaucoma (POAG) is the most common form of glaucoma, and its etiology is unknown. It has been known since 1997 that mutation of the myocilin gene (also known as TIGR for trabecular meshwork-inducible glucocorticoid response) can cause juvenile-onset open angle glaucoma (JOAG) [2]. More than 70 mutations in the myocilin gene contribute to the pathogenesis of approximately 3% of familial autosomal dominant adult-onset open angle glaucoma and a greater proportion of JOAG [3]. Among the identified myocilin gene mutations, the Pro370Leu mutation (OMIM 601652; allelic variant .007) is responsible for one of the most severe glaucoma phenotypes [4-7].

Myocilin is a 504 amino acid secreted glycoprotein that is normally expressed in a number of ocular and nonocular tissues; a high amount of the protein is detectable in the trabecular meshwork (TM), which is involved in the regulation of intraocular pressure (IOP) [8,9]. Myocilin is known to influence the biological and physiological properties of human trabecular meshwork (HTM) cells and tissue, implicating the protein in intracellular matrix, extracellular matrix (ECM), and cell-ECM functions [10,11]. However, these or other roles for mycocilin have not been confirmed. The wild-type form of myocilin induces changes in stress fibers, focal adhesions of HTM cells, and is involved in interactions with ECM molecules and matricellular signaling-like activities [9-14]. In expression studies, myocilin has been observed to colocalize with structures that are part of the secretory pathway including the endoplasmic reticulum (ER), Golgi apparatus, and intracellular vesicles [14-16], and associates with mitochondria and cytoplasmic filaments [13,14].

The significance of mutant forms of mycilin in the pathogenesis of glaucoma remains elusive. It has been suggested that the myocilin gene mutation that causes POAG represents a gain of function mechanism [17-19]. A series of functional assays for mutant myocilin have shown that mutant proteins become insoluble in the detergent Triton X-100 and nonsecretory, inhibiting secretion of the normal myocilin protein [14,15,20,21]. Pro370Leu mutant myocilin may represent a misfolded, highly aggregation-prone form of the protein, which accumulates in the ER of HTM cells, resulting in abnormal HTM cells morphology and cell death [16]. The Tyr437His mutant mouse model of open-angle glaucoma has demonstrated that Tg mice exhibit pathological changes similar to phenotype in glaucoma patients, with the mutated myocilin being nonsecreted and accumulated in HTM cell cytoplasm, similar to the aforementioned cell culture experiments [22]. Both in vitro and in vivo data strongly indicate that the accumulation of mutant myocilin in the ER of HTM cells is necessary for development of myocilin-associated glaucoma [15,16,20-22]. The influence of myocilin gene mutations, in particular Pro370Leu, on the ER are unclear and need to be investigated.

In eukaryotic cells, the ER is responsible for the synthesis, modification, and delivery of proteins to their proper target sites [23]. ER alterations can alter protein folding, leading to the accumulation of aberrant proteins in the ER. This, in turn, activates a signaling response termed the ER stress response, which includes induction of ER-resident molecular chaperones and foldases to complete the folding process, downregulation of the biosynthetic load of the ER through the shut-off of protein synthesis, and increased clearance of unfolded proteins from the ER through the upregulation of ER-associated degradation (ERAD) [23,24]. There are multiple implications of ER stress response in health and disease. Under ER stress, aberrantly folded proteins can activate the ER stress response to return the ER to its normal physiological state. When this process does not remedy the stress situation, pathology can result [24-26]. Indeed, the accumulation of misfolded protein in ER caused by gene mutations is most likely the central event in the initiation of cell death in some diseases including autosomal-dominant diabetes and familial Alzheimer's disease-linked presenilin-1 (PS1) and the alternatively spliced form of the presenilin-2 gene lacking exon 5 (PS2V)-induced cell apoptosis by effect on ER stress response [27-31]. ER and mitochondria collaborate in both physiological and pathophysiological cell functions, indicating that the gateways to cell death involve complex and coordinated interactions between these two organelles [24,32].

Accumulation of mutant myocilin proteins in the ER of HTM cells is pathogenic and induces cell death [15,16]. However, knowledge of the molecular mechanism underlying the cellular toxicity of mutant myocilin and the link with ER is scant. The present study was undertaken to further clarify the molecular details of the role of Pro370Leu mutant myocilin. We hypothesized that Pro370Leu mutant myocilin is misfolded and compromises the ER as well as other organelles function, leading to HTM cell dysfunction or death. Here, we report the results of our examinations of the effect of Pro370Leu mutant myocilin on the ER stress response and mitochondrial membrane potential (*Δψm*) of HTM cells.

## Methods

### Human trabecular meshwork cell culture

The eyes from 5 human donors were obtained from the Eye Bank of Zhongshan Ophthalmic Center within 24 h of death due to automobile accidents. After enucleation, all eyes were cut equatorially behind the ora serrata. The ciliary body, iris, and lens were removed, and the trabecular meshwork was dissected under a microscope (X40). Excised trabecular meshwork explants were placed at 37 °C in Dulbecco's modified Eagle's medium (DMEM), supplemented with with 10% fetal bovine serum, 50 mg/ml penicillin, and 50 units/ml streptomycin. Outgrowth of cells was observed after 1-3 weeks. Phase contrast microscopy confirmed that confluent cultures formed a monolayer with the typical morphological characteristics of cultured human trabecular meshwork cells. HTM cells were collected with 0.25% trypsin for 1-2 min. HTM cells derived from 22-, 25-, 29-, 30-, and 32-year-old healthy donors were cultured using a technique described by Polansky et al. [33]. HTM cells were cultured in Dulbecco's Modified Eagle's Medium (Gibco, Grand Island, NY) supplemented with 10% fetal bovine serum, 50 units/ml penicillin, and 50 mg/ml streptomycin. For experiments, the fourth- or fifth-passage HTM cells were plated in six-well tissue culture dishes and allowed to grow to subconfluence.

### Plasmid construction

The full-length myocilin cDNA clone has been described by Wang and Johnson [34]. *Bam*HI and *Eco*RI restriction enzyme cleavage site were added to the 5' and 3' end of the myocilin cDNA by PCR. The obtained fragment was cloned into a mammalian expression plasmid (pcDNA3.1[+]; Invitrogen, Carlsbad, CA). We produced Pro370Leu mutations with the QuikChange site-directed mutagenesis kit (Stratagene, La Jolla, CA), according to the manufacturer's instructions. The specific PCR primers for Pro370Leu mutagenesis were as follows: 5'-CTA CCA CGG ACA GTT CCT GTA TTC TTG GGG TGG CTA-3', 5'-TAG CCA CCC CAA GAA TAC AGG AAC TGT CCG TGG TAG-3'. The sequence of Pro370Leu mutant myocilin plasmid was verified with the DNA sequencer to confirm both the correct insertion and absence of undesirable mutations.

### Cell transfection and induction of endoplasmic reticulum stress

Fourth- or fifth-passage HTM cells grown to about 80-85% confluence were transiently transfected with about 1.5 to 2.0 μg wild-type or Pro370Leu mutant myocilin expression plasmids using LipofectAMINE^TM^ (Invitrogen, Carlsbad, CA) according to the manufacturer's instructions. After 6 h, the cells were washed once with culture medium, and further cultured for 40 h. The efficiency of transfection was estimated in cells transiently transfected with the pcDNA3.1(+)/GFP vector by counting the percentage of GFP-positive cells using flow cytometry analysis. The transfection efficiency was about 38-43%. HTM cells were transfected with pcDNA3.1(+) plasmid and used as a negative control. To induce ER stress, HTM cells were stimulated with 2.5 μg/ml tunicamycin (Tm; Sigma, St. Louis, MO), and cultured for 6 h. The control dishes were treated without Tm.

### Reverse transcription polymerase chain reaction

Total cellular RNA was extracted from HTM cells using TRIzol reagent (Invitrogen Corp.) according to the manufacturer's instructions. Purified RNA was reverse- transcribed with SuperScriptTM One-Step RT-PCR System (Invitrogen Corp.) using a GeneAmp PCR (Perkin Elmer, Shelton, CT). For a semiquantitative analysis of the PCR product, we used a normalization procedure according to the manufacturer's instructions. We performed 30 cycles and used an annealing temperature of 50 °C and 55 °C, respectively, for GRP78 and GAPDH. Primer pairs for the gene-specific RT-PCR analyses were as follows: human GRP78, forward primer 5'-GAC ATC AAG TTC TTG CCG TT-3' and reverse primer 5'-CTC ATA ACA TTT AGG CCA GC-3', and human GAPDH, forward primer 5'-CGT ATT GGG CGC CTG GTC ACC-3' and reverse primer 5'-GGG ATG ATG TTC TGG AGA GCC C-3' [35,36]. The sizes of the RT-PCR products were 260 bp and 587 bp, respectively. Each experiment was repeated three times. The density of GAPDH was used as an internal standard to normalize densitometry measurements of the gel bands. Semiquantitative analysis was performed by Gel-Pro Analyzer software. The statistical difference was analyzed by the Student's t test. Value was expressed as the mean±standard deviation (SD), and was calculated from three independent experiments. A p value less than or equal to 0.05 was considered statistically significant.

### Western blot analysis

After treatment, HTM cells were washed with cold phosphate buffered saline (PBS; pH 7.4) and lysed in 100 μl protein extraction reagent (Pierce, Rockford, IL). After boiling for 5 min, the cell lysates were centrifuged at 4 °C for 10 min, then the supernatants were collected and stored at -70 °C. Each sample (20 μg) was loaded onto 12% SDS-polyacrylamide gels and electrophoresed at 100 V for 1 h. The resolved proteins were transferred electrically to PVDF membranes and incubated with 0.5% skim milk in tris-buffered saline containing 0.05% Tween 20 (TTBS) for 1 h. The membrane was probed with goat polyclonal GRP78 antibody (1:500 final dilution, Santa Cruz Biotechnology Inc., Santa Cruz, CA), rabbit antiphospho-eIF2α antibody (1:1000 final dilution, Cell Signaling Technology, Inc., Beverly, MA), rabbit eIF2α antibody (1:1000 final dilution, Signaling Technology, Inc.), and developed with Phototope-HRP Western Blot Detection System kit (Cell Signaling Technology, Inc.) according to the manufacturer's instruction. As an internal control, the levels of β-actin were examined on the same blot at the same time. Each experiment was repeated three times. Quantitative analysis of western blotting was performed using Gel-Pro Analyzer software. The statistical difference of the data was determined by the Student's t test.

### Measurement of *Δψm*

To measure the *Δψm* of HTM, we used the fluorescent probe JC-1 (5,5',6,6'-tetrachloro-1,1',3,3'-tetraethylbenzimidazole carbocyanide iodide; Molecular Probes, Eugene, OR) and followed a method described in reference [37]. Briefly, HTM cells (5x10^5^) were collected by trypsinization, washed in PBS, then incubated with 1.0 μg/ml JC-1 for 15 min at 37 °C. Cells were pelleted at 200x g for 5 min, and washed in PBS, then analyzed immediately by flow cytometry analysis, using a BD FACS Aria^TM^ flow cytometer and BD FACSDiVa software (Becton Dickinson, Mountain View, CA). For complete depletion of *Δψm* (positive control), the mitochondrial uncoupler, carbonyl cyanide 3-chlorophenylhydrazone (CCCP; 50 μM), was used. Photomultiplier settings were adjusted to detect green fluorescence (λ_em_=525 nm) of JC-1 monomer on the filter 1 (FL-1 detector) and the red fluorescence (λ_em_=590 nm) of JC-1 aggregates on the filter 2 (FL2 detector). In each experiment, at least 10,000 events were analyzed. The relative aggregate:monomer (red:green) fluorescence intensity values were used for data presentation. The statistical difference of the data was determined by the Student's t test.

## Results

### Effect of Pro370Leu mutant myocilin on the expression of glucose-regulated protein78

**Figure 1:**
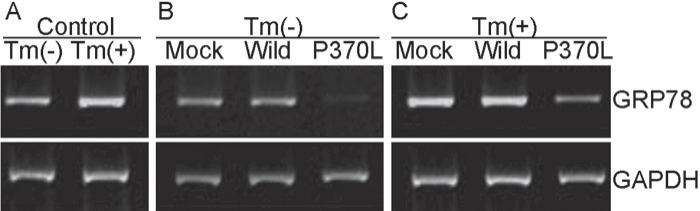
Expression of glucose-regulated protein 78 mRNA in human trabecular meshwork cells. **A**: Primary cultured human trabecular meshwork (HTM) cells were either exposed to 2.5 μg/ml Tm for 6 h or not treated. **B**: Primary cultured HTM cells were transiently transfected with the indicated expression plasmids. **C**: Primary cultured HTM cells were transiently transfected with the indicated expression plasmids, then cells were exposed to 2.5 μg/ml Tm for 6 h. Total RNA was isolated from each cell population, and subjected to RT-PCR for glucose-regulated protein78 (GRP78) mRNA (upper panels) or GAPDH mRNA (lower panels). The gel was representative of three separate experiments. The following terms were used: mock, cells transfected with pcDNA3.1(+) expression plasmid; wild, cells transfected with wild-type myocilin expression plasmid; and P370L, cells transfected with Pro370Leu mutant myocilin expression plasmid.

**Figure 2:**
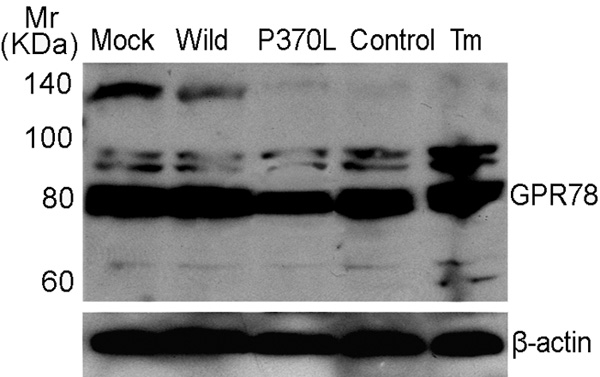
Expression levels of glucose-regulated protein78 protein in human trabecular meshwork cells. Human trabecular meshwork (HTM) cells were transfected with each expression plasmid (pcDNA3.1(+), wild-type, Pro370Leu mutant myocilin plasmids) or were treated with 2.5 μg/ml Tm. Untreated cells served as the control. Western blot was performed with an anti-glucose-regulated protein78 (GRP78) antibody and an anti-β-actin antibody. The blots are representative of three experiments. The following terms were used: mock, cells transfected with pcDNA3.1(+) expression plasmid; wild, cells transfected with wild-type myocilin expression plasmid; and P370L, cells transfected with Pro370Leu mutant myocilin expression plasmid.

The wild-type and Pro370Leu mutant myocilin plasmids were transiently transfected into HTM cells for 48 h. The overexpression levels of the wild and mutant myocilin mRNA were similar, which increased approximately sixfold compared with that of control by semiquantitative PCR analysis (data not shown). The 78 kDa glucose-regulated protein (GRP78) is known to be a key marker of ER stress. When HTM cells were stimulated with 2.5 μg/ml Tm for 6 h to induce ER stress via prevention of protein glycosylation, the level of GRP78 mRNA significantly increased compared with that of control (Figure 1A). Correspondingly, GRP78 mRNA in HTM cells expressing Pro370Leu mutant myocilin exhibited a significant decrease (65.5±2.0%, mean±SD, n=3, p less than or equal to 0.05) compared to cells transfected with wild-type myocilin plasmid (Figure 1B). To confirm that Pro370Leu mutant myocilin specifically inhibited GRP78 mRNA induction, we transiently transfected HTM cells with each expression plasmids and then treated the cells with Tm for 6 h. GRP78 mRNA was markedly reduced (62.7±1.4%, mean±SD, n=3, p less than or equal to 0.05) in Pro370Leu-mutant-transfected HTM cells compared with wild type-myocilin-transfected HTM cells (Figure 1C). The expression of GAPDH mRNA as an internal control didn't change in any of the cell populations. Furthermore, similar expression levels of GRP78 protein in the transfected HTM cells were confirmed using western blot analysis. GRP78 protein expression was decreased by 22.5±2.3% (mean±SD, n=3, p less than or equal to 0.05) in HTM cells expressing Pro370Leu mutant myocilin compared to transfectants of wild-type myocilin plasmid (Figure 2), whereas there was no significant difference in GRP78 protein expression between wild-type-myocilin-transfected and pcDNA3.1(+)-transfected cells.

### Altered activation of phospho-eIF2α by Pro370Leu mutant myocilin

**Figure 3:**
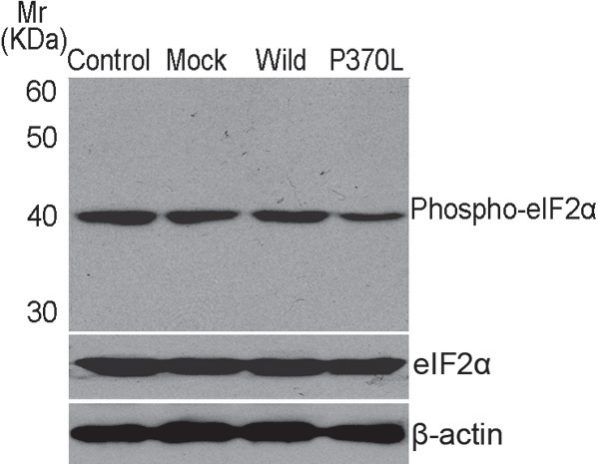
Altered activation of phospho-eIF2α by Pro370Leu mutant myocilin. The phosphorylation of eIF2α was reduced in human trabecular meshwork (HTM) cells transiently transfected with Pro370Leu mutant myocilin plasmid. The total eIF2α protein is shown to demonstrate equal loading of lanes. β-Actin was used as an internal control. The blots are representative of three experiments. The following terms were used: mock, cells transfected with pcDNA3.1(+) expression plasmid; wild, cells transfected with wild-type myocilin expression plasmid; and P370L, cells transfected with Pro370Leu mutant myocilin expression plasmid.

To further investigate the effect of Pro370Leu mutant myocilin on the regulation of ER stress response, we analyzed the activation of phosphorylaton of the α subunit of eukaryotic translation initiation factor 2 (eIF2α). Under ER stress, the outcome of phosphorylation of eIF2α is the attenuation of protein translation [24,38]. Western blot analysis of HTM cells using an anti- phospho-eIF2α antibody revealed the constitutive expression of phospho-eIF2α protein in untreated HTM cells and in HTM cells transfected with wild-type myocilin or pcDNA3.1(+) plasmids (Figure 3). In HTM cells expressing Pro370Leu mutant myocilin the level of phospho-eIF2α protein was markedly diminished (30.6±2.6%, mean±SD, n=3, p less than or equal to 0.05) compared with HTM cells transiently transfected with wild-type myocilin plasmid, although the total quantity of eIF2α was not appreciably altered (Figure 3).

### Analysis of *Δψm* in human trabecular meshwork cells

**Figure 4:**
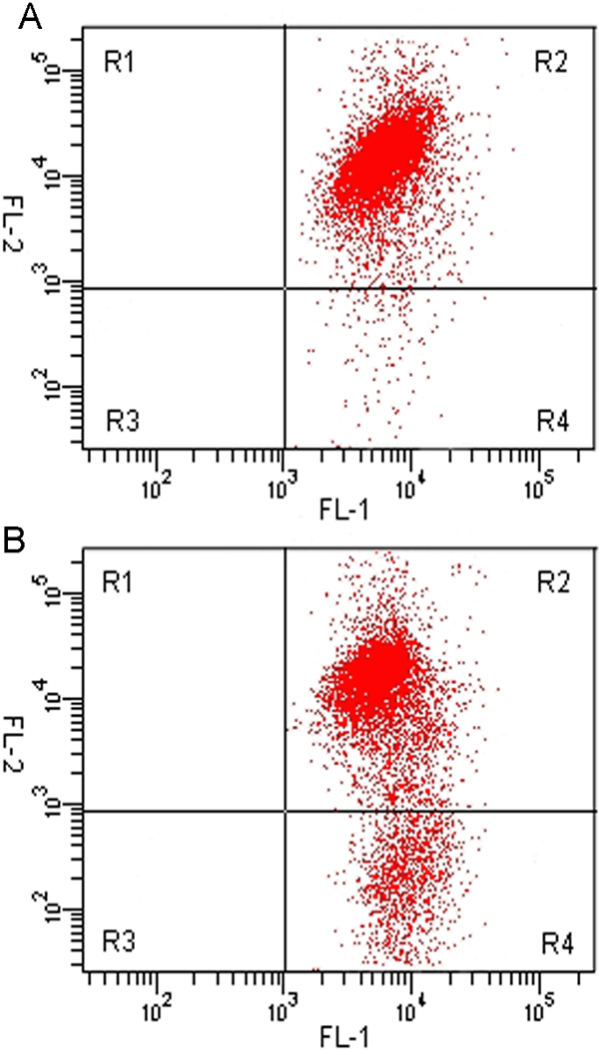
Analysis of *Δψm* in human trabecular meshwork cells. **A**: The scattergram showed the most cells presented in the R2 region with high red fluorescence (FL-2) and high green fluorescence (FL-1), which revealed normal *Δψm* of human trabecular meshwork (HTM) cells. **B**: HTM cells transfected with Pro370Leu mutant myocilin plasmid. The cells markedly decreased in the R2 region, indicating that Pro370Leu mutant myocilin induced loss of *Δψm*. A representative experiment of three is shown.

**Figure 5:**
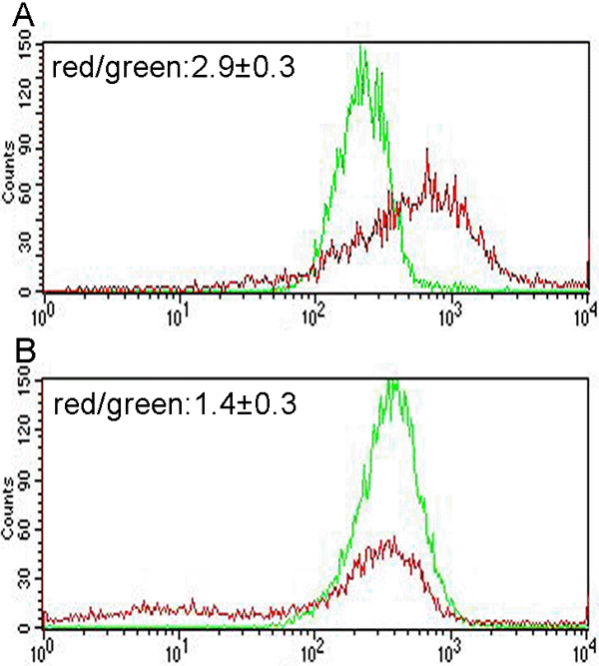
Quantification of *Δψm* in human trabecular meshwork cells by JC-1. A total of 5x10^5^ normal and transfected human trabecular meshwork (HTM) cells with Pro370Leu mutant myocilin plasmid were analyzed by flow cytometry. **A**: Normal HTM cells. **B**: HTM cells transfected with Pro370Leu mutant myocilin plasmid. Red fluorescence (Red line) decreased in Pro370Leu-transfected HTM cells. The reduction in the ratio of Red:green JC-1 fluorescence indicates the drop of *Δψm*.

To understand the effect of Pro370Leu mutant myocilin on mitochondria function of HTM cells, we analyzed the change of *Δψm* by flow cytometry analysis with the fluorescent probe JC-1. JC-1 is selectively taken up into mitochondria and so is a reliable indicator of change in *Δψm* [37,39]. At hyperpolarized *Δψm*, JC-1 forms J aggregates in a rapidly reversible manner, emitting red fluorescence. During depolarization of mitochondria, JC-1 leaks and consequently reduces dye concentration in the mitochondrial matrix, and emits a green fluorescence [40]. Ratiometric measurements of the red to green JC-1 fluorescence indicates *Δψm*. Presently, the staining pattern of JC-1 for normal HTM cells was established as the standard. The scattergram (Figure 4A) revealed that the highest number of cells presented in the R2 region with high red fluorescence (FL-2) and high green fluorescence (FL-1). When the HTM cells were transfected with Pro370Leu mutant myocilin plasmid, the cells markedly decreased in the R2 region, whereas the cell population of the transfected cells increased in the R4 region (Figure 4B). The change was the consequence of a marked reduction in the percentage of Pro370Leu-mutant-transfected HTM cells with normal *Δψm* (mean±SD, 69.2±10.3% versus 92.5±12.7%; n=3, p less than or equal to 0.05). Furthermore, the quantitative studies showed the same results. The HTM cells expressing Pro370Leu mutant myocilin had a lower ratio of red to green JC-1 fluorescence than normal HTM cells (mean±SD, 1.4±0.3 versus 2.9±0.3; n=3, p less than or equal to 0.05; Figure 5). The *Δψm* of HTM cells transfected with wild-type myocilin or pcDNA3.1(+) plasmids were similar to normal HTM cells (data not shown).

## Discussion

Missense mutations in myocilin that accumulate in large aggregates in the ER of HTM cells lead to cell death [15,16]. However, the molecular mechanism causing the cellular toxicity of mutant myocilin is still unknown. This study provides evidence that the Pro370Leu mutation in the myocilin gene, which is known to produce severe disease phenotypes [4-7], downregulates the signaling pathway of the ER stress response. Under ER stress, genetic expression of ER molecular chaperones is immediately induced, in which GRP78 is a central regulator [24,41]. To determine the association between Pro370Leu mutant myocilin and the ER stress response, we analyzed the expression of GRP78 in HTM cells. We found the expression of GRP78 in HTM cells is attenuated by Pro370Leu mutant myocilin. This finding was unexpected, given the knowledge that mutant myocilins, including G364V and Q368X, concentrate into punctate aggregates in the ER of HTM cells and upregulate the expression of GRP78 [15]. We suggest the possible reason for the discrepancy between the previous and our present results relates to differences in experimental conditions such as methods of cell culture, and the myocilin mutants studied. GRP78 induction under pathological conditions may represent a major cellular protective mechanism for cells to survive ER stress and thus has implications in organ preservation [41,42]. Our findings indicate that the Pro370Leu mutant myocilin downregulates the ER stress response and the subsequent inhibition of GRP78 induction may weaken the protein-folding machinery that normally detects and corrects misfolded proteins. This downregulation of the ER stress response by Pro370Leu mutant myocilin might perturb the protective mechanism, thus increasing the vulnerability of HTM cells to ER stress. Such a mechanism is similar to the pathogenic role of mutant PS1, which is linked to familial Alzheimer Disease on the signaling pathway of unfolded protein response [30,31].

Our results indicate that Pro370Leu mutant myocilin inhibits phosphorylation of eIF2α. During ER stress the phosphorylation of eIF2α plays an important role in attenuating protein translation through the inhibition of the assembly of the 80S ribosome [43,44]. In Eif2s1^tm1Rjk^ mutant mice, the reduction in eIF2α phosphorylation leads to beta-cell failure and type 2 diabetes. Phosphorylation of eIF2α is necessary to prevent ER dysfunction and insulin secretion disorder [45]. Therefore, we propose that the Pro370Leu mutant myocilin-mediated loss of eIF2α phosphorylation increases the translation of misfolded myocilin, which exceeds the ER folding capacity, damages the structural functional integrity of the ER, and predisposes HTM cells to failure. Alternatively, because translation attenuation is partially defective in HTM cells with Pro370Leu mutant myocilin, misfolded myocilin might accumulate in the ER rather than being transported to organelles, such as the Golgi apparatus, plasma membrane, or ECM, which could have an impact on its appropriate cellular/extracellular function sites. Moreover, cells respond to ER stress by changing gene expression, selectively translating specific genes that encode survival proteins through the phosphorylation of eIF2α [43,44]. The production of the Pro370Leu mutant myocilin inhibits the phosphorylation of eIF2α in the HTM cells, which could impair this adaptive response and cell survival. Details concerning the signaling regulation of ER stress response and the function of missense-mutated myocilin remain to be elucidated.

ER and mitochondria cooperate with each other in the control of physiological cell function like metabolism, calcium signaling, and in pathophysiological situations such as cell death pathways [24,32]. In this study, we performed an analysis to determine whether mitochondria play a role in the HTM cell injury that is caused by Pro370Leu mutant myocilin. We measured the change of *Δψm* in transfected HTM cells, because *Δψm* is a highly sensitive indicator of the energetic state of mitochondria and is an important monitor of key cellular processes [39,40,46]. Flow cytometry analysis with the fluorescent probe JC-1 revealed a decline of *Δψm* in HTM cells transfected with a plasmid encoding Pro370Leu mutant myocilin. Indeed, the depolarization of mitochondria may be an early sign of mitochondrial dysfunction and may precede many other signs of cell injury [39,47]. We suggest that Pro370Leu mutant myocilin damages the mitochondrial function of HTM cells; collapse of *Δψm* probably causes disruption of the outer mitochondrial membrane, depletion of ATP, and the release of damage factors. This remains to be confirmed, as does the Pro370Leu mutant myocilin-mediated mitochondrial dysfunction of HTM cells.

Earlier studies have shown that the pathologic characteristics of glaucoma include an increase in the accumulation of extracellular material in the TM and a loss of TM cells. Our present experiments demonstrate that Pro370Leu mutant myocilin downregulates GRP78, inhibits the expression of phosphorylation of eIF2α, and affects the ER stress response. We suggest that the disturbed response may prolong ER stress and change the secretory function of HTM cells, leading to an altered extracellular environment and, ultimately, to HTM cell dysfunction and death. Myocilin is localized to the mitochondria of HTM cells, and dexamethasone treatment of HTM cells increases myocilin-mitochondria association; moreover, patients with POAG display the greatest mitochondrial DNA sequence alterations [12,13,46]. However, the relationship, if any, between mutant myocilin and mitochondrial function has not been reported. In the present study, we showed that Pro370Leu mutant myocilin disturbs the *Δψm* of HTM cells. Hence, we propose that mitochondrial dysfunction may be a risk factor for HTM cell death caused by Pro370Leu mutant myocilin.

In conclusion, the present data from our research demonstrate that Pro370Leu mutant myocilin disturbs the protection to ER stress and mitochondria function. We suggest that Pro370Leu mutant myocilin causes perturbation of the ER stress response and mitochondria dysfunction through a gain of function, which is likely to render HTM cells more vulnerable to secondary challenges such as oxidative stress and mechanical stretch, resulting in cell dysfunction or death. Our study provides a framework for related mechanisms for multiple mutations of myocilin. Such a mechanism in glaucoma might compromise the function of TM, increase outflow resistance of aqueous humor, and ultimately lead to the disease. This present study provides new insights into the relationship between myocilin-associated glaucoma and the function of the ER and mitochondria.
